# Medical Advertising

**Published:** 1875-09

**Authors:** 


*Medical Advertising.*

Dr. Edgar, in his address before the Medical Editors' Association, has taken a bold, outspoken course that must meet the approval of every physician who loves his profession, and desires to see it occupy the high position to which it is entitled. His remarks on the recklessness with which diplomas are scattered broadcast over the land, and his denunciation of that suicidal course that is making *us* the laughing-stock of Europe, and our *diplomas* rather something to be ashamed of than an object of pride, deserve the thanks of his brother physicians. We regret our want of space prevents a full report of his address, but the following remarks, giving as they do the key-stone of the existing state of things, are too true to be left out. He says:

"There is another kind of advertising which has reached a point in America which threatens the humiliation of the profession, almost beyond redemption. I allude to the multiplication of medical schools. It is well known to the profession that many of these schools are organized solely to advertise the men composing the faculties, seeing this is a method not interdicted by the code of ethics.

"A dozen, or fifteen or twenty doctors, more or less, meet and organize themselves into a medical faculty, a charter is procured and the chairs 'fanned out' among them, and circulars scattered thick as blackbirds in autumn, heralding the wonderful advantages of this new school; a card is in all the publications of the city, secular and scientific. Thus no means are spared to make known that Prof. A. has the chair of obstetrics, and Prof. B. that of surgery, etc., to the end of the list;* then come as many more names (with their addresses) as assistant lecturers, waiting to don the discarded mantles of their superiors. In this way twenty-five or thirty doctors are advertised into practice, over their neighbors, often every way their superiors, (except, perhaps, the teacher of chemistry, who has to be *paid*, as the public have little use for his wares.)

"Other professional men, observing the success of this shrewd trick to inveigle the public, and seeing no other way to keep even in the race, organize another school, and the community is startled by the announcement of as many more professors in medicine born in a day. Thus a city of a hundred thousand inhabitants may boast two, or perhaps three medical schools, equipped and in full blast, with large classes, where fifty properly qualified students it might be hard to find.

"By 'beating the brush' in all directions, and offering all admission who apply, with or without education, with or without money or price, in this way are large classes collected; and if the graduating list of either school is not quite satisfactory (on account of the nine months' short cut) there is generally timber enough near at hand, none the worse for having been used, half a dozen or more 'ad eundems' are added, and the bill is filled, the effect upon the public satisfactory, which of course is the end sought.

"But what effect on the profession has the conduct of these schools? The means used by one party to secure pupils, being regarded as dishonorable and unfair by their competitors, personal differences are begotten which extend to all the rings or cliques composing the faculties. In many instances so intense is the hatred thus engendered, that it is shared by the friends of the doctors in the several schools, thus filling the social atmosphere with its pestiferous blight; and any physician who may have the temerity to express his opinion concerning these great advertising frauds upon the profession and public, is at once ostracised by the ag-

---

*And some of these professors, not content with all this advertising, manage to have the secular press notice their every movement; if they leave town for a day or a year, it is chronicled in the local of the daily; any operation they perform, of importance, the reporter hears of in time to be present and report, thus utterly ignoring, if not defying, the code of ethics.

grieved faculty, amounting to a system of black-mailing, and ever thereafter treated as a personal enemy. This discord farther finds expression in the conduct of medical societies, in efforts to rule or ruin the same; in combined and well concerted efforts to ruin the reputation of physicians whose only crime is that they are not of their party. It intrudes itself into the private chambers of the sick, no less than the medical management of all public charities and sanitary boards; creating divisions where the profession should be a unit; finally, the social status of the profession in the communities cursed with these rings, is so disgraceful that every intelligent and right-minded physician feels compromised, and ready to rue the day of his identification with a profession thus dishonored. Neither is this all: indeed the worst effect remains to be told, that which humiliates and breaks down the claim of the profession to rank with the learned professions, viz: the sending forth yearly, if not *oftener*, hundreds of young men, certified by diplomas to be competent to assume the responsibilities of a medical and surgical practice, when they have not the first qualification for the duties of either."—*St. Louis Medical and Surgical Journal.* H. B. L.

